# An improved model based on the support vector machine and cuckoo algorithm for simulating reference evapotranspiration

**DOI:** 10.1371/journal.pone.0217499

**Published:** 2019-05-31

**Authors:** Mohammad Ehteram, Vijay P. Singh, Ahmad Ferdowsi, Sayed Farhad Mousavi, Saeed Farzin, Hojat Karami, Nuruol Syuhadaa Mohd, Haitham Abdulmohsin Afan, Sai Hin Lai, Ozgur Kisi, M. A. Malek, Ali Najah Ahmed, Ahmed El-Shafie

**Affiliations:** 1 Department of Water Engineering and Hydraulic Structures, Faculty of Civil Engineering, Semnan University, Semnan, Iran; 2 Department of Biological and Agricultural Engineering, Zachry Department of Civil Engineering, Texas A&M University, College Station, Texas, United States of America; 3 Department of Civil Engineering, Faculty of Engineering, University Malaya, Kuala Lumpur, Malaysia; 4 Faculty of Natural Sciences and Engineering, Ilia State University, Tbilisi, Georgia; 5 Institute of Sustainable Energy (ISE), Universiti Tenaga Nasional (UNITEN), Selangor, Malaysia; 6 Institute of Energy Infrastructure (IEI), Universiti Tenaga Nasional (UNITEN), Selangor, Malaysia; Newcastle University, UNITED KINGDOM

## Abstract

Reference evapotranspiration (ET_0_) plays a fundamental role in irrigated agriculture. The objective of this study is to simulate monthly ET_0_ at a meteorological station in India using a new method, an improved support vector machine (SVM) based on the cuckoo algorithm (CA), which is known as SVM-CA. Maximum temperature, minimum temperature, relative humidity, wind speed and sunshine hours were selected as inputs for the models used in the simulation. The results of the simulation using SVM-CA were compared with those from experimental models, genetic programming (GP), model tree (M5T) and the adaptive neuro-fuzzy inference system (ANFIS). The achieved results demonstrate that the proposed SVM-CA model is able to simulate ET_0_ more accurately than the GP, M5T and ANFIS models. Two major indicators, namely, root mean square error (RMSE) and mean absolute error (MAE), indicated that the SVM-CA outperformed the other methods with respective reductions of 5–15% and 5–17% compared with the GP model, 12–21% and 10–22% compared with the M5T model, and 7–15% and 5–18% compared with the ANFIS model, respectively. Therefore, the proposed SVM-CA model has high potential for accurate simulation of monthly ET_0_ values compared with the other models.

## Introduction

### Background

Reference evapotranspiration (ET_0_) is a fundamental variable in irrigation management. Estimation of ET_0_ is essential to determining the timing, amount, frequency and scheduling of irrigation [1]. Considering water scarcity and the need to increase food production, knowledge of ET_0_ at different time scales is central to water management [2] and to planning and management of water resources [3]. Because the physical processes in the hydrological cycle are complex, statistical models are often applied to model these processes [4]. In addition to regression and statistical methods [5], artificial intelligence methods have been used in modelling of ET_0_. The reasons for use of intelligence methods such as neural networks or fuzzy logic include the ability to perform quick and easy calculations, deliver high accuracy and handle large volumes of data [6]. However, artificial intelligence methods can have complex architectures and structures, which make simulation difficult [7]. Regression techniques and the support vector machine (SVM) have simpler structures but unknown parameters [8]. The different models applied in the simulation offer both advantages and disadvantages. The following sections present the main methods and their potential future uses.

The artificial natural network generates a nonlinear relationship between the driven inputs and output based on one black box. The model is highly complex, although it has a high ability to simulate soft computing for different fields such as evaporation [3,9]. One of the complexities of this method is related to the numerous parameters required for the artificial neural network (ANN) that should be determined based on numerous sensitivity analyses, trial and error experiments, and number of neurons, and the type of transfer function should be computed based on accurate computations. The ANN considers multiple layers in the computation, each of which requires accurate determination of details. Thus, decision makers face many unknown parameters and a complex structure. One important question related to this method is what type of neural network to use. Various studies have reported different types of neural networks, such as radial or multi-layer perception [9,10]. For example, Kumar et al. [10] applied various architectures of ANN for simulation of evaporation. The radial neural network yielded the best results for the simulation of evaporation, and the number of layers and neurons were computed based on a trial and error process. Additionally, Kisi et al. [9] applied the generalized regression neural network to simulation of evaporation, and a nonlinear regression theory was added to the ANN as an estimation function to improve the training level and test level for the algorithm. The method was based on minimization of squared error, and investigation of the results showed that the generalized regression neural network could decrease the root-mean-square error (RMSE) by between 20 and 32% compared with the radial neural network [9]. However, when the ANN method is compared with metrological methods, the ANN might have better performance. Metrological methods such as the application of lysimeters are related to the soil condition and crop condition. These methods are complex and require expensive tools, and furthermore, they are advanced for specific aims and cannot be used in all problems [9].

Another method in the simulation field is genetic programming (GP), which consists of evaluation programming that can receive input data and generate a nonlinear relationship among the data to compute the outputs [1,2]. The GP method considers a random search of the decision space based on a tree structure and uses the tree structure based on the mathematical presentation of relationships [1,2]. The random set of trees is considered for the first iteration of simulations, and the best trees are subsequently selected based on the value of the objective function. The objective function can be considered in terms of an error index such as RMSE. The selected trees are subsequently modified based on different operators such as mutation and crossover [1–3]. Thus, accurate determination of the value of mutation and crossover requires sensitivity of analysis. The decision variables for this method are inserted based on the definition of an initial population of chromosomes. Therefore, sensitivity analysis is considered for determination of the initial population of chromosomes to yield the best results for the simulation. One advantage of GP is related to application of the mathematical function and arithmetic operators such that the method can find an accurate relationship between the input data and output data. Therefore, the GP has a high mathematical ability to find the best relationship between the input and output data, whereas such mathematical operators have not been defined for other methods in the simulation fields. The importance of this method is high for simulation of ET0 because ET0 depends on different climatological parameters based on a nonlinear relationship, and the determination of the best equation between inputs and output is difficult.

One of the other methods used in the simulation of evaporation is related to tree models. Tree models divide the data into sub-regions and consider the tree application of the data of each sub-region. The input space was divided into sub-regions, a fit linear model was used in each sub-region, and the regression tree was built for the data for this level [8,9]. Certain reports show that this method requires large amounts of sampling input data for simulation of evaporation, which is one of the potential disadvantages of the method. Although the method was considered to be straightforward, reports have shown that it can encounter problems with large numbers of data.

SVM is known as a good tool for evaporation methods. The method has a simple structure, but the unknown parameter is one of the disadvantages of these methods. Kisi [3] computed monthly evaporation based on SVM, a tree model and GP. The study showed that the values of the unknown parameters of this method were obtained based on trial and error, as in many previous studies. One innovation in the development of this method is related to preparation of the method with accurate parameter values. Optimization tools can be powerful compared with trial and error processes such that the unknown values are inserted into the optimization algorithms, and the accurate values of these parameters are computed based on minimizing error indices such as RMSE.

In general, soft computing and regression methods have high capability for ET0 simulation, although each method contains disadvantages as well as advantages. Such methods can produce better performance than empirical models because empirical models require large amounts of data and different data, meaning that certain data might not be available for decision makers, and soft computing and regression methods can simulate the results with less computational time.

Guven and Kisi [11] used GP, neural networks and empirical models to calculate monthly ET_0_ using average temperature, number of sunshine hours and relative humidity as inputs. The results showed that the neural network model had RMSE values that were lower by 10–12% relative to the GP model and by 10–20% relative to the empirical models. The applied GP contains parameters such as mutation and crossover, and the accurate values of these parameters were computed based on the variation of value of the objective function versus the parameter values such that the best value of mutation and crossover should minimize the error indices such as RMSE and MAE.

Cobraner [12] used two different fuzzy methods to calculate the ET_0_ of a station in the USA using maximum temperature, sunshine hours, relative humidity and wind speed as inputs. The correlation coefficient between ET_0_ simulated using the subtractive clustering-based fuzzy methods and the observed data was higher than that simulated by the grid partition-based fuzzy neural network and empirical models. The type of membership function used in this study was triangular, and the types of functions were computed based on trial errors. Different types of membership functions were used, and the function that could minimize the objective function was considered the best function.

Karmialdinit et al. [13] used neural networks, GP, and support vector methods for simulation of ET_0_ using sunshine hours, relative humidity and average temperature. The neural network method reduced the RMSE by 10–15% compared with the support vector and by 8–10% compared with the GP method. A sensitivity analysis was considered for commutation of the random parameters of GP, the different neural networks were tested on a case study, and the radial basis was selected because the results of this method matched with the observed data to a high degree.

Tabari et al. [14] used the neural network, fuzzy neural network and GP to calculate the monthly ET_0,_ and showed that the fuzzy neural network method had significantly less error than the neural network and GP.

Samui [15] simulated monthly ET_0_ using SVM with radial, polynomial and sigmoid kernel functions and showed that the mean error produced by the radial kernel function was less than those obtained with the polynomial and sigmoid kernel functions. Additionally, the correlation coefficients of the radial kernel were higher, by approximately 5–7% and 6–9% compared with the polynomial and sigmoid kernels, respectively. The different applied kernel functions and unknown parameters were computed based on trial and error, and although trial and error is a standard method, it was not an accurate tool.

Ladlani et al. [16] used the regression neural network, GP, tree model and empirical models to calculate the monthly ET_0_ using maximum temperature, minimum temperature, wind speed and number of sunshine hours. The regression neural network method led to a more consistent correlation with the observations than the GP and empirical models.

Kisi [17] found that the evolutionary neural network method had lower RMSE values than the multi-layer neural network and regression for simulating the monthly ET_0_ in arid and desert regions. Additionally, Kim et al. [18] used an improved neural network and genetic algorithm for simulating the monthly ET_0_ using temperature, sunshine hours, relative humidity and wind speed as inputs. The genetic algorithm was used to calculate the number of hidden layers as well as the weights in the neural network. The results showed that the RMSE for the improved neural network was 10–12% lower than those of the SVM and regression models.

Citakoglu et al. [19] used the adaptive-network-based fuzzy system (ANFIS) method, multi-layer neural network and GP to simulate the monthly ET_0_ at two stations in Turkey under a moderate climate. The results showed that the ANFIS had lower RMSE, by 10–12% and 13–15% than the neural network and GP, respectively. Malik and Kumar [20] used an improved neural network method with the particle swarm algorithm (PSA) and a GP method to calculate the monthly ET_0_ for a station in Turkey. The results showed that PSA reduced RMSE by 12–15% relative to the GP method. Kim et al. [21] used a multi-layer neural network, regression and GP to calculate the ET_0_ using the number of sunshine hours, relative humidity, temperature and wind speed at several stations in Korea. The results showed that the multi-layer neural network method had higher correlation with the observations than regression and GP. Keshtgear et al. [22] used ANFIS and tree models to calculate the monthly ET_0_ using a combination of temperature, sunshine hours, wind speed and relative humidity for a station in Iran. The results showed that ANFIS had a higher correlation coefficient and lower error than the tree model. Deo et al. [23] used a spline method, tree model and SVM to simulate monthly ET_0_ using temperature, sunshine time, and wind speed and found that SVM had lower RMSE values than the tree and spline models. Mehdizadeh et al. [24] used multivariate adaptive regression spline (MARS), SVM with a polynomial kernel, SVM with a radial kernel, and gene expression programming (GEP) to simulate the ET_0._ The results showed that the MARS and SVM with a radial kernel performed better than the SVM with a polynomial kernel and GP methods.

### Problem statement and innovation

From the background, it could be summarized that the chronological advancement process for a prediction model for ET0 is still in progress. Notwithstanding the up-to-date progression in developing a prediction/estimation model for ET_0_, achievement of an accurate estimation/prediction model for ET_0_ is still an essential subject for hydrologists, irrigation managers and agriculture experts. During the most recent decades, many efforts have been directed to this critical point of research. In this context and with respect to our survey, few empirical and closed form formulations have been developed to accurately estimate the ET_0_ value, but these formulations required several climatological parameters to be available for estimation of the ET_0_. The fact that certain of these climatological parameters are unavailable at several locations in the world has motivated experts to investigate other methods as an alternative to these empirical methods. Recently, data-driven models and machine learning models such as ANN, ANFIS and SVM have been introduced, and among these methods, SVM was identified as the best method for successfully supplying the best accuracy of ET_0_ values. However, according to recent studies on implementation of SVM models, the main drawback of this model is the optimization values for the internal parameters, including ε, C, and σ. These parameters reflect the same commonality of successions and can be computed via learning experience. Therefore, this drawback has motivated researchers to integrate the SVM with an advanced optimization model to optimally estimate these SVM parameters rather than using the traditional trial and error procedure. However, difficulty still exists in developing such integration due to the clash, conflict or lack of harmony between the mathematical procedure of the SVM and the optimization algorithms. Furthermore, those optimization algorithms that are able to be integrated with SVM still experienced problems such as trapping during search for the global optima and time consumption for the convergence rate. Therefore, researchers are motivated to use nature-inspired optimization algorithms to solve these internal parameters of the SVM model.

Based on this information and in line with the logical chronological advancement process in model development for ET_0_ prediction/estimation, a novel modelling structure is proposed that integrates the classical SVM (as predictor) with one of the most recent nature-inspired optimization algorithm, namely, the cuckoo algorithm (CA). In the current research, the CA algorithm is used to optimize the internal parameters of the SVM method, with the advantage of a fully optimized model for effective feature mapping and prediction to overcome the abovementioned drawbacks in the existing methods.

The current study improves the SVM by optimizing its parameters using a new optimization algorithm, the cuckoo algorithm (CA), for simulation of the monthly ET_0_. Many previous studies have reported on the SVM used in this work based on a trial and error method [25], a method that has a simple structure and can be a good choice for decision makers. However, it is necessary to further develop the method. One strategy is to compute the unknown parameter values for the SVM based on optimization algorithms. The unknown values for the optimization algorithms were considered as decision variables, and an error index was considered as the objective function. The values of the parameters should be computed such that the value of the error index or objective function reaches the lowest value. This process is based on artificial intelligence and iteration cycles. The important innovation of this paper is related to the development of the SVM method based on an optimization algorithm. The CA used in this study was selected to develop the SVM method because when the optimization tool is listed, factors such as rapid speed and ease of computation should be considered for the optimization algorithm, and the method should not become trapped in the local optimum to solve the optimization problem without an issue. Ming et al. [26,27] recommended the CA as a powerful method for water resource management problems, citing the high flexibility of the method with different boundary conditions for hydraulic and hydrologic problems and its rapid computation and simple structure as important features of the method. Thus, the SVM method, which is useful for simulation of evaporation, and the CA, which is a good tool for optimizing the selection of the SVM parameters, were used in this study.

### Objective

The main objective of this study is to propose a prediction model that can accurately predict the ET_0_. In addition, we introduce a new model structure by integrating the classical predictor, which is the SVM in this study, with the cuckoo algorithm (CA), which is considered one of the most recent nature-inspired optimization algorithms. The proposed model was compared with the actual ET_0_ values attained from the experimental models. Furthermore, with respect to the previous literature review, it has been shown that the GP, ANFIS and M5T models are useful and successful methods for predicting ET_0_, and therefore, they are selected for comparison with the proposed improved method. To examine the proposed model, the actual ET_0_ data and actual climatological parameters for a monitoring station in India are used.

## Methodology

### Support vector machine (SVM)

The SVM method is one of the most successful and widely used methods in hydrological simulations [24,25]. The linear SVM equation can be expressed as follows:

$$f\left(x\right)=w^{Tr}x+b$$ (1)

where *x* is the input variable, *w* are the weight coefficients, b is the bias, Tr is the transposition and f(x) is the estimated target. SVM attempts to reduce the difference between the observed and simulated values. Thus, SVM simulates based on minimizing the objective function, which is an error index. The optimization process is defined as follows [25]:

$$minimize\frac{1}{2}||w|{|}^{2}+c\sum_{i=1}^{m}\left(\xi_{i}^{-}+\xi_{i}^{+}\right)$$ (2)

$$Subject\;\left(to\right)\left(w\;x_{i}+b\right)-y_{i}<\varepsilon +\xi_{i}^{+}$$

$$y_{i}-\left(w_{i}x_{i}+b\right)\leq \varepsilon +\xi_{i}^{-})$$ (3)

where C is the penalty coefficient, m is the number of training data, $\xi_{i}^{-}$ and $\xi_{i}^{+}$are the violations of the data whose different values are greater than ξ the permitted range with observable value), and $w_{i,}x_{i},y_{i}$ are the weight of the variables, the input variable, and the target observation variable, respectively. The values of w and b are calculated using Eqs 2 and 3 and are subsequently substituted into Eq 1. Different kernel functions are used in the SVM method, and the radial kernel function is widely used and effective in various hydrological simulations and water resource management [24,25]. Thus, Eq 1 is considered as follows:

$$f\left(x\right)=w^{Tr}.K\left(x,x_{i}\right)+b$$ (4)

$$K\left(x,x_{i}\right)=exp\left(-\frac{x-x_{i}}{2\gamma^{2}}\right)$$ (5)

Where $K\left(x,x_{i}\right)$ is the kernel function, and *γ* is the parameter of the kernel function. The parameters with unknown values for the SVM method (including, C and *ε*) are entered into the optimization as decision variables in the algorithm. The purpose of the hybrid SVM and CA is to find the exact value of the parameters and simulate ET_0_ via the SVM. The SVM simulates ET_0_ based on receiving the input to the problem, and the value of the parameters, C and *ε* are modified based on the CA. The SVM is used as a direct tool for ET_0_ simulation, the CA is subsequently used as an indirect tool based on a mathematical operator, and artificial intelligence is used to find the accurate values for the parameters of the SVM to obtain the best results.

### Cuckoo algorithm

The CA is based on the life of the cuckoo bird. The egg laying and growth of the cuckoo is the main basis of the algorithm. Some birds avoid the trouble of nesting and parental duties by destroying one egg from a host bird and leaving a cuckoo egg for the host bird to raise. Cuckoos accomplished this task by mimicking the colour and pattern of eggs in each nest such that the cuckoo egg is similar to the actual eggs of the host. Therefore, the cuckoo egg in the nest has the opportunity to grow and survive. The cuckoos used in this model are of two types: adult cuckoos and eggs. The pseudo-code of CA includes the following steps [24]:

1. Determine the initial habitat of the cuckoos (the initial response);
2. Assign a few eggs to each cuckoo;
3. Determine the egg laying radius (ELR) for each cuckoo by the number of eggs and the distance to the destination using the following equation:
  $$ELR=\alpha \times \frac{Number\left(of\right)current\;(cuckoo)egg}{Total\left(of\right)eggs}\times \left(var_{h_{i}}-var_{low}\right)$$ (6)
  Where ${var}_{h_{i}}$ is the maximum decision variable, ${var}_{low}$ is the minimum value of the decision variable and α is the number that controls ELR;
4. Cuckoo lays eggs in its respective ELR range;
5. Remove eggs using poor objective function;
6. Determine the value of the objective function for each adult cuckoo;
7. Limit the maximum number of creatures in the environment;
8. Group the cuckoos and determine the superior habitat;
9. Migration of cuckoos to the superior habitat at this stage. In every move towards the target habitat, the cuckoo does not travel the full distance but only λ % of the path via the φ deviation of the radian. For each cuckoo, λ is a number between zero and one, and φ is a random number between -ω,ω. The value of *ω* is approximately 0.530.

### Hybrid of support vector machine and cuckoo algorithm (SVM-CA)

**Fig 1:**
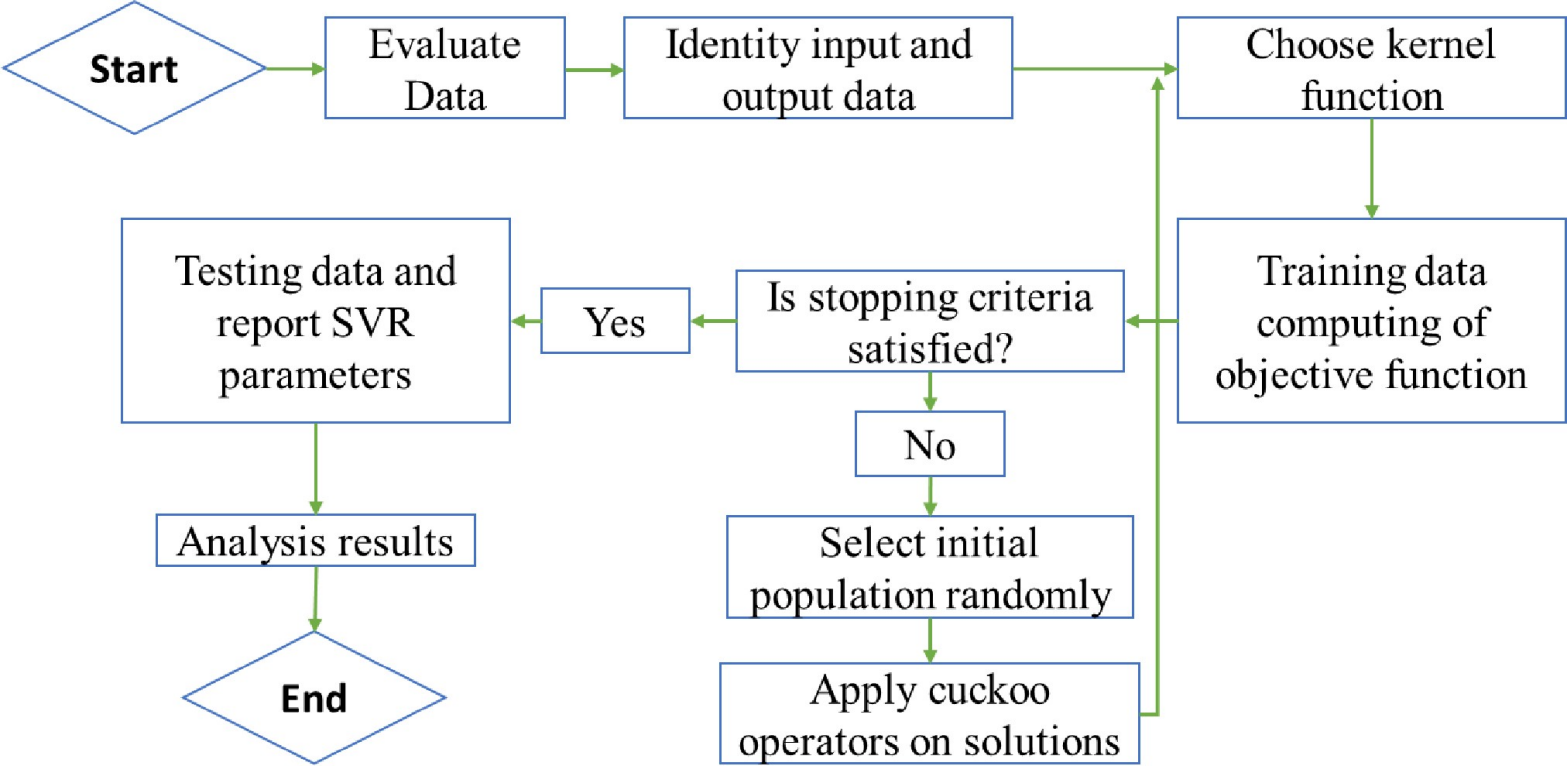
Hybrid structure of SVM and CA.

SVM has unknown parameters, and thus the following steps are used to upgrade the method:

1. The γ, C, and *ϵ* parameters are initialized in SVM;
2. The effective inputs for the potential ET_0_ value are determined based on the correlation coefficient;
3. Seventy percent of the data are used in training, 15% are used in the validation period and 15% are used in testing;
4. The objective function of the current study is the RMSE;
5. The stop condition for the algorithm is checked. If it is satisfied, go to step 6; otherwise, go to step 7;
6. The validation and test steps are applied, and the optimal values of SVM method coefficients are noted;
7. The values of unknown parameters of the SVM method are entered as the initial population in CA;
8. Steps 1–9 covered in Section 2.2 are applied to the initial cuckoo's habitat or the unknown values of the parameters based on CA. At this point, the process returns to Step 2. Fig 1 shows the stages of the hybrid structure. First, the unknown values of the SVM parameters are inserted into the algorithm as the initial population. The value of each parameter is allocated to each egg, such as the box. Thus, the SVM method begins with a random value for the unknown parameters, and the objective function (i.e., the RMSE index as a useful and known index) is computed. It is natural at the launch of the algorithm for the RMSE to have a high value such that the other levels of the algorithm attempt to improve the results. When the objective function is computed, a certain number of eggs (as the value of the parameters of the SVM method that obtain the highest value for the RMSE) are eliminated as the worst value for these parameters. The values of the unknown parameters are considered as decision variables for the algorithms. The solutions that remain after the elimination of solutions are improved based on the migration operator with the following equation, which can help to generate the new solutions or new values for the unknown parameters that can be used in continuation of the algorithm.

$$POP_{i}^{new}=POP_{i}^{current}+\beta \left(POP^{best}-POP_{i}^{current}\right)$$ (7)

Where ${POP}_{i}^{new}$ is the new solution or value of the parameters, ${POP}^{best}$ is the best solution, current solution and β is the movement coefficient. Eq 6 is applied because the decision maker for the optimization problem can choose a limited number of eggs to keep the search space from becoming crowded. The input data for the ET_0_ simulation are inserted into the SVM, and the SVM method simulates the ET_0_ based on a random value of unknown parameters. These parameter values are determined based on the initial population of the cuckoo and eggs.

### M5 model tree (M5Tree)

The decision tree displays a series of rules that lead to a category or quantity. The model is notably simple and can simulate a large number of attributes and high dimensions. Two main levels can be observed for the model. The first level is related to division of the search space into sub-regions, and the second level is related to generation of data using the information of each sub-region. The new trees are built based on the received input data in the sub-region.

Deciduous trees are formed into a series of separate groups via sequential separation of data to increase the separation of groups [23]. The structure of a tree model consists of the root, the inner nodes and the leaf. An inference algorithm or a division criterion is used to produce a decision tree. The division criterion for the model tree is the standard deviation of the class values that arrive at each node as quantities of error, and the model calculates the expected reduction in the error as a result of the test of each attribute at that node. The standard deviation reduction (*SDR*) is calculated as follows:

$$SDR=sd\left(T\right)-\sum \frac{\left|T_{i}\right|}{\left|T\right|}sd\left(T_{i}\right)$$ (8)

where *T* is a series of samples that reach the node, *Ti* is the samples that are the i-th output, and *sd* denotes the standard deviation. After maximizing all possible derivations, the M5 tree selects an attribute that maximizes the expected reduction. The division further develops the structure of a large parietal tree, which results in better fitting. To overcome the problem of fitting, the tree must be pruned by replacing a tree with a leaf. Therefore, the second stage in the design of a tree model involves pruning of the grown tree and replacing the trees with linear regression functions. This tree modelling technique divides the space of the input parameters into smaller areas or sub-spaces, and in each of these, a tree regression model is fitted.

### Genetic programming (GP)

**Fig 2:**
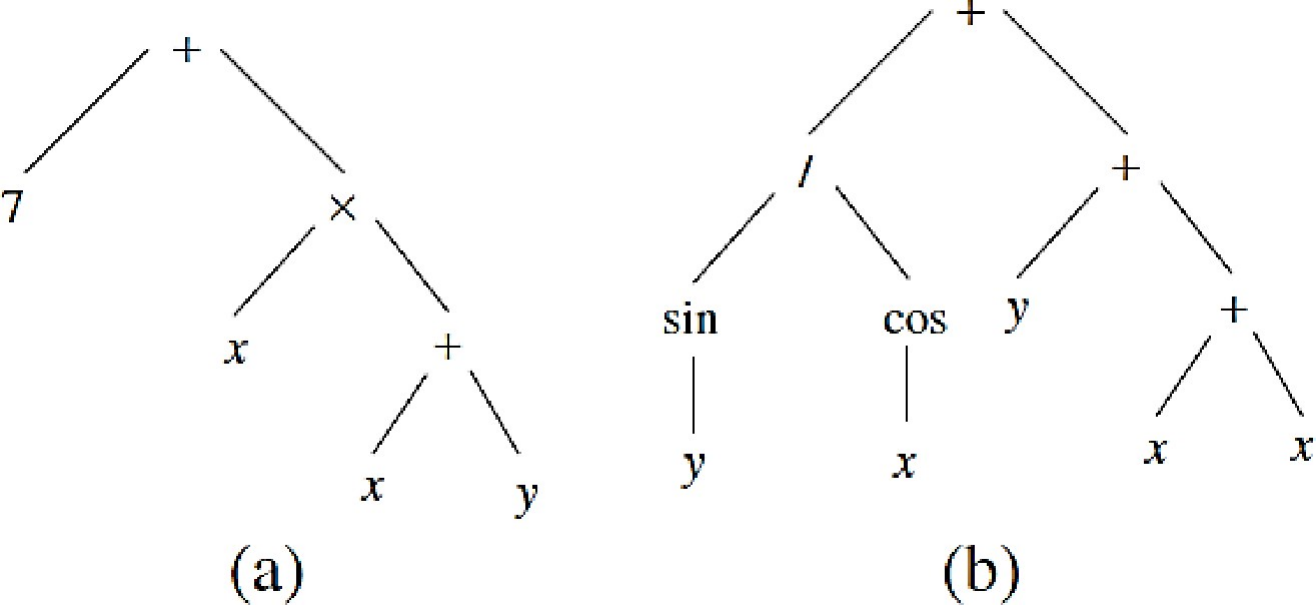
Structure of genetic programming (GP).

**Fig 3:**
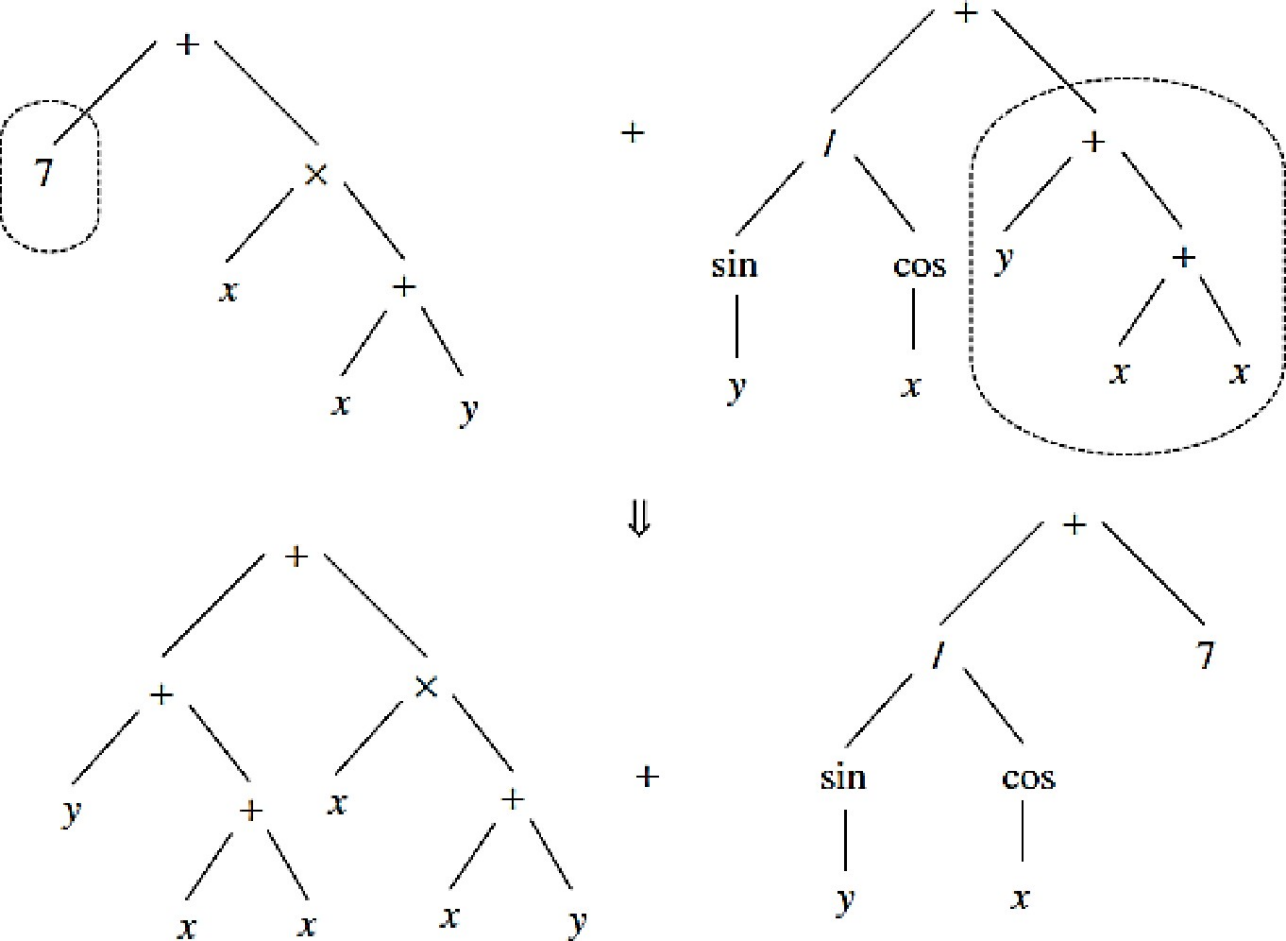
Crossover operator for genetic programming (GP).

**Fig 4:**
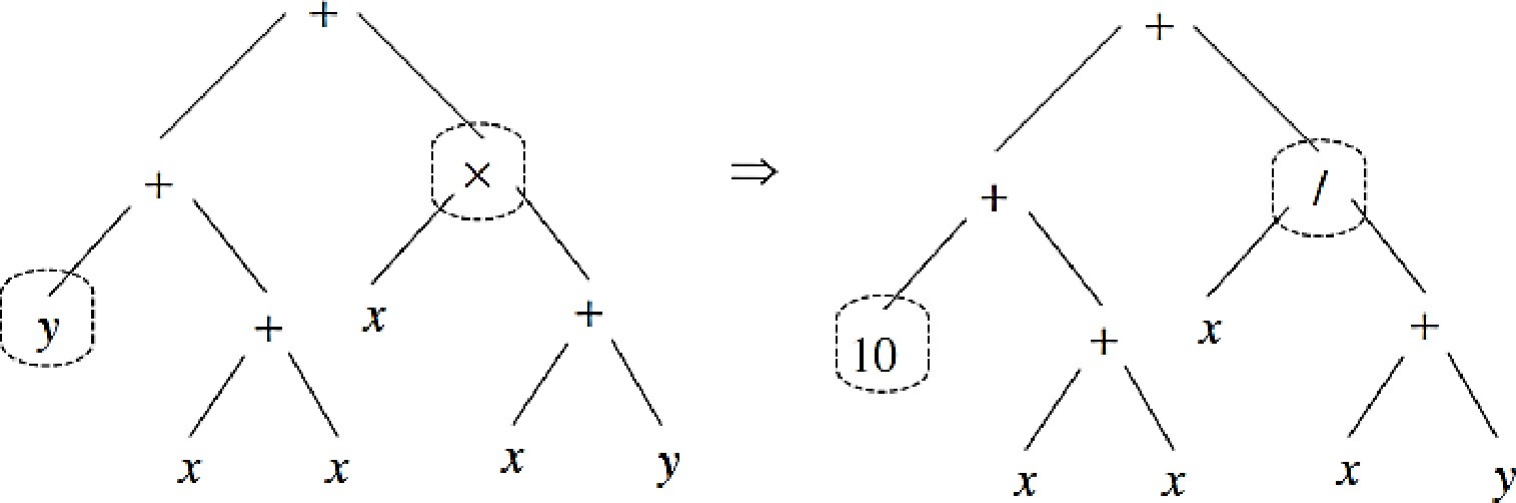
Mutation operator.

GP is a method commonly used in hydrological simulation and water resource management, is based on search and iteration, and includes the following steps [24]. GP is a random heuristic search that acts based on natural evolution and tree structure. The main advantage of this method is the application of many functions and variables, which results in a high ability for simulation problems. First, many trees or solutions are considered to randomly generate an initial population. The nature of the problem affects the selection of the size or the number of trees. Each tree includes a mathematical, logical equation; numerical and non-numerical variables; arithmetic operators (±,×,÷); and mathematical functions (e.g., sines and cosines). A simple mathematical tree and the variables and mathematical operators are shown in Fig 2A and 2B shows a more complex illustration of the tree, including a number of nodes and branches. Selected numbers of trees are considered for generation of new trees. The objective function or error index, such as RMSE, is computed for each tree, and the best trees with the lowest error index values are selected for generation of new trees for the next levels. Two operators known as the crossover and mutation operators are applied to the solutions. Sweeping random sub-trees based on the initial trees are created, and the crossover operator is shown in Fig 3. The mutation operator is applied in the GP such that the random node or function is exchanged with a random one. Fig 4 shows the mutation operator. The process continues until the stop criteria is satisfied.

1. The GP method consists of two sets. The first set is known as the terminal set, e.g., $T=\left[x,1,2,,-1,-2,\ldots \right]$ The second set is known as the functions set, which can be equivalent to F={÷,×,+,−,exp,sinus,gocinus,log,…}. The X in the terminal set is the input variable. The method searches the best functions with the best arithmetic operators to generate a nonlinear relationship between the inputs and output;
2. A set of random initial responses is considered from sets F and T;
3. This step is intended to calculate the objective function for each chromosome and, if necessary, it can apply penalty functions and add them to the value of the objective function;
4. This step is devoted to the application of genetic operators, including mutation and crossover;
5. The iteration and performance steps continue to the extent that the value of the objective function reaches a fixed value. The optimal or near optimal value is created as the solution to the problem. In the current study, arithmetic operators $\left\{+,-,\times ,÷\right\}$ and mathematical functions $\left\{\sqrt{x},\sqrt[{3}]{x,}x^{2},x^{3},\text{ln}\left(x\right),e^{x},\sin\left(x\right),\cos\left(x\right),A\;rctan\left(x\right)\right\}$

are used.

### Adaptive neuro-fuzzy inference system (ANFIS) method

Although the neural network has high capability, it still contains certain deficiencies when used in simulation. Neuro-fuzzy systems are applied in simulation of hydrological variables by combining the semantic transparency of the rules of systems founded on the learning capacity of neural networks [21,22]. The combination of neural and fuzzy networks can reduce the computational time and error rates and is a better match for the problem under consideration. A fuzzy neural system is presented in Fig 2 in which the circle represents a fixed node, and the square symbolizes a matching node. Two inputs x and y and an output z are considered for convenience. The fuzzy systems include several layers for simulation of results. The fuzzy rules are known as the Sugeno rules or models that attempt to simulate the output results. The decision maker should attempt to determine the architecture of the method and layers such that the method uses the different membership functions for generation of a nonlinear relationship between the input and output data.

The Sugeno model used in this study is one of the most popular models among different fuzzy systems. Sugeno's first-order system operates based on the following rules:

$$Rule\left(1\right)=if\left(x\right)is\left(A_{1}\right)\;and\left(y\right)is\;\left(B_{1}\right),then\;\left(Z_{1}\right)=p_{1}x+q_{1}y+r_{1}$$ (9)

$$Rule\left(2\right)=if\left(x\right)is\left(A_{2}\right)\;and\left(y\right)is\;\left(B_{2}\right),then\;\left(Z_{2}\right)=p_{2}x+q_{2}y+r_{2}$$ (10)

where *A* and *B* are the fuzzy sets and *p*, *q* and *r* are the design parameters that are calculated during the training period.

Layer 1: The nodes in the first layer generate the membership degrees of an input variable that belongs to the appropriate fuzzy sets using membership functions. Each node contains adaptive nodes:

$$O_{i}^{1}=\mu_{A_{i}}\left(x\right)$$ (11)

$$O_{i}^{1}=\mu_{B_{i}}\left(y\right)$$ (12)

Where $\mu_{B_{i}}$ and $\mu_{A_{i}}$ are the node membership functions. The parameters of this layer are known as premise parameters.

Layer 2: Layer 2 contains fixed nodes that are represented by the symbol ⊓ in Fig 2. The output of each node is the multiplication of all of the input signals to that node:

$$O_{2,i=}W_{i}=\mu A_{i}\left(x\right)\mu B_{i}\left(y\right)$$ (13)

Where $W_{i}$ is the output for each node.

Layer 3: The third layer consists of fixed nodes, which are identified by N in Fig 2. The nodes in this layer are the normalized outputs of Layer 2, which are calculated based on the following equation:

$$O_{3,i}=\bar{w_{l}}=\frac{w_{i}}{w_{1}+w_{2}}$$ (14)

Layer 4: Each node in Layer 4 is associated with a node function:

$$O_{4,i}=\bar{w_{l}}f_{i}=\bar{w_{l}}\left(p_{i}x+q_{i}y+r\right)$$ (15)

Where $\overset{-}{w_{l}}$ is the normalized fire power of Layer 3. The parameters *p*, *q* and *r* in this layer are known as consequent parameters.

Layer 5: Layer 5 consists of a node that calculates the output by summing the input values:

$$O_{5i}=\sum \bar{w}f_{l}=\frac{\sum w_{i}f_{i}}{\sum w_{i}}$$ (16)

**Fig 5:**
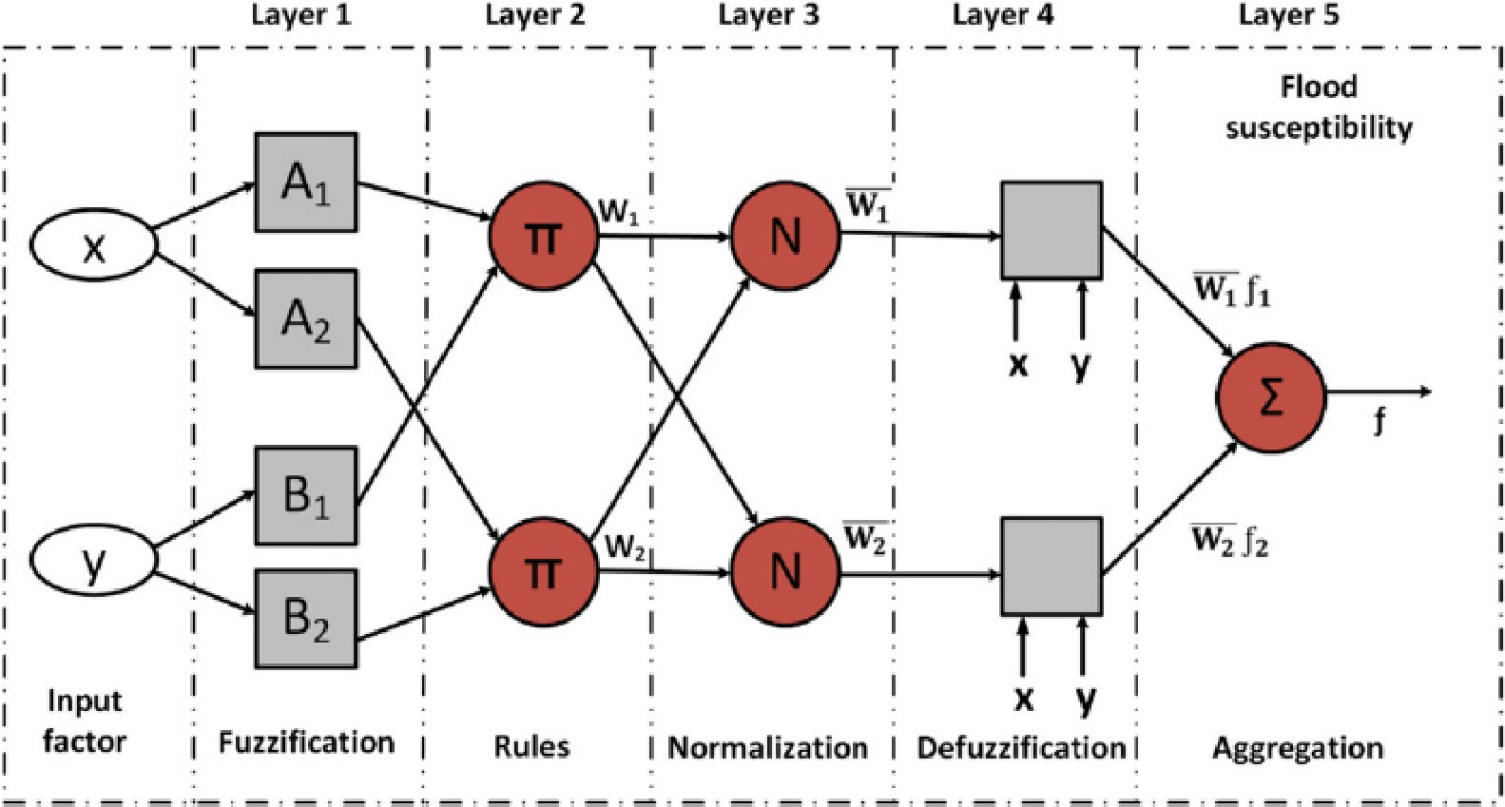
ANFIS structure.

ANFIS uses a hybrid learning algorithm that includes a combination of descending gradients for calculation of the premise parameters and the least squares method for determining the consequent parameters. The main task of the algorithm is to learn, set up and accurately calculate the consequent and premise parameters to prepare ANFIS for simulation (Fig 5).

### Empirical models

Direct methods for calculating ET_0_ use certain parameters that are difficult to calculate. Contrary to direct methods, the United Nations Food and Agriculture Organization’s FAO-56PM method contains equations that are commonly used to calculate the ET_0_ [22,23,25]. Allen et al. [28] proposed the FAO-56PM method as a standard methodology used to evaluate other empirical and mathematical models:

$$ET_{0-PM}=\frac{0.408\left(R_{n}-G\right)+\gamma \left(\frac{900}{T+273}\right)u_{2}\left(e_{s}-e_{a}\right)}{\Delta -\gamma \left(1+0.34u_{2}\right)}$$ (17)

The current study considered the application of three models found to be appropriate in other studies as empirical models [26,27]. Eq 17 relates to the calculation of the evaporation method by Priestley-Taylor [29]:

$$ET_{0-PT}=\frac{\alpha }{\lambda }\frac{\Delta }{\Delta +\lambda }\left(R_{n}-G\right)$$ (18)

Eq 18 relates to the Hargreaves calculation [28]:

$$ET_{0-H}=0.0135R_{s}\left(T_{mean}+17.8\right)$$ (19)

Eq 19 relates to the Makkink calculation [30]:

$$ET_{0-MH}=0.70\frac{\Delta }{\Delta +\gamma }\frac{R_{s}}{\Delta }$$ (20)

where R_n_ is the net radiation (MJm^-2^day^-1^), G is the soil heat flux density (MJm^-2^day^-1^), R_S_ is the solar radiation (MJm^-2^day^-1^), T_mean_ is the average temperature (centigrade) at a height of 2 m, u_2_ is the wind speed at 2 m (ms^-1^), e_s_ is the saturation vapour pressure (kPa), $e_{a}$ is the real vapour pressure, α is a constant coefficient, λ is the evaporated heat (MJkg^-1^) and Δ is the vapour pressure curve slope. The wind vane at the station measures the wind direction, and a cup anemometer is used to measure the wind velocity. The maximum and minimum thermometer are used to record the maximum and minimum temperature, respectively, and the monthly temperature is considered as the average asthmatic of the mean daily temperature. The humidity (in unit percent) is measured by a hydrographer. Sunshine is measured using a sunshine recorder.

## Case study

**Fig 6:**
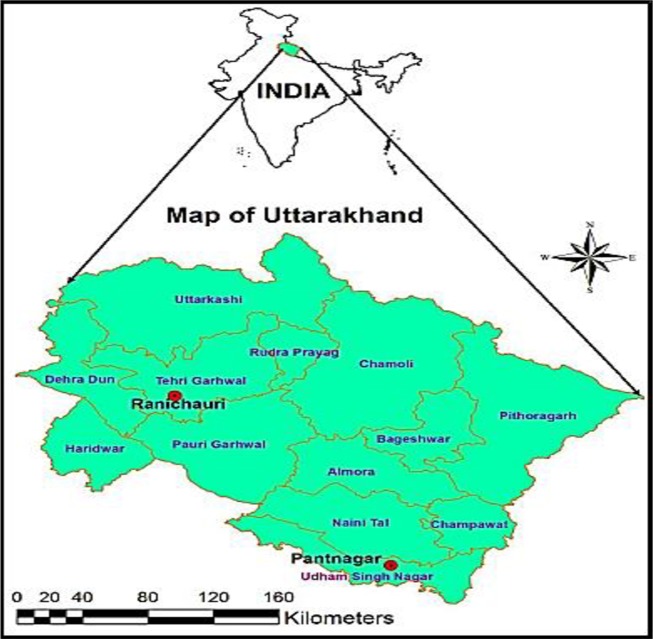
Location of the basin.

This study deals with a station in Pantangar, India, located at (79^0^38’0”, 29^0^0’0”), as shown in Fig 6. The station is located in the central Himalayan area of India, and experiences an average rainfall of 1,400 mm per year. Information collected from a weather center site in the area was used to simulate monthly ET. This included $T_{min,}T_{max}$ (maximum and minimum temperature), *RH*_*1*,_ *RH*_*2*_ (relative humidity; the *RH*_*1*_ was recorded at 7 AM and *RH*_*2*_ was recorded at 2 PM), *S*_*w*_ (wind speed), H_ss_ (sunshine hours) and ${EP}_{m}$ (monthly ET_0_). Meteorological tools were considered to collect data at a meteorological observatory or weather station. There are many weather stations in India, which are regulated by Indian meteorological department. The meteorological data were obtained from weather stations in the current study. Previous hydrological studies consider three levels for the investigating of models [1,2]. The first level is known as training level to prepare the method and obtain the parameters and structure of method. The second level is related to the validation, and the third level is related to the test level so that the ability of models are determined based on the application of the model on the data of this period. The longer period is allocated to the training level as the decision maker can prepare the method well and then the remaining of periods are used for the verification and calibration levels [1,2,8].

**Fig 7:**
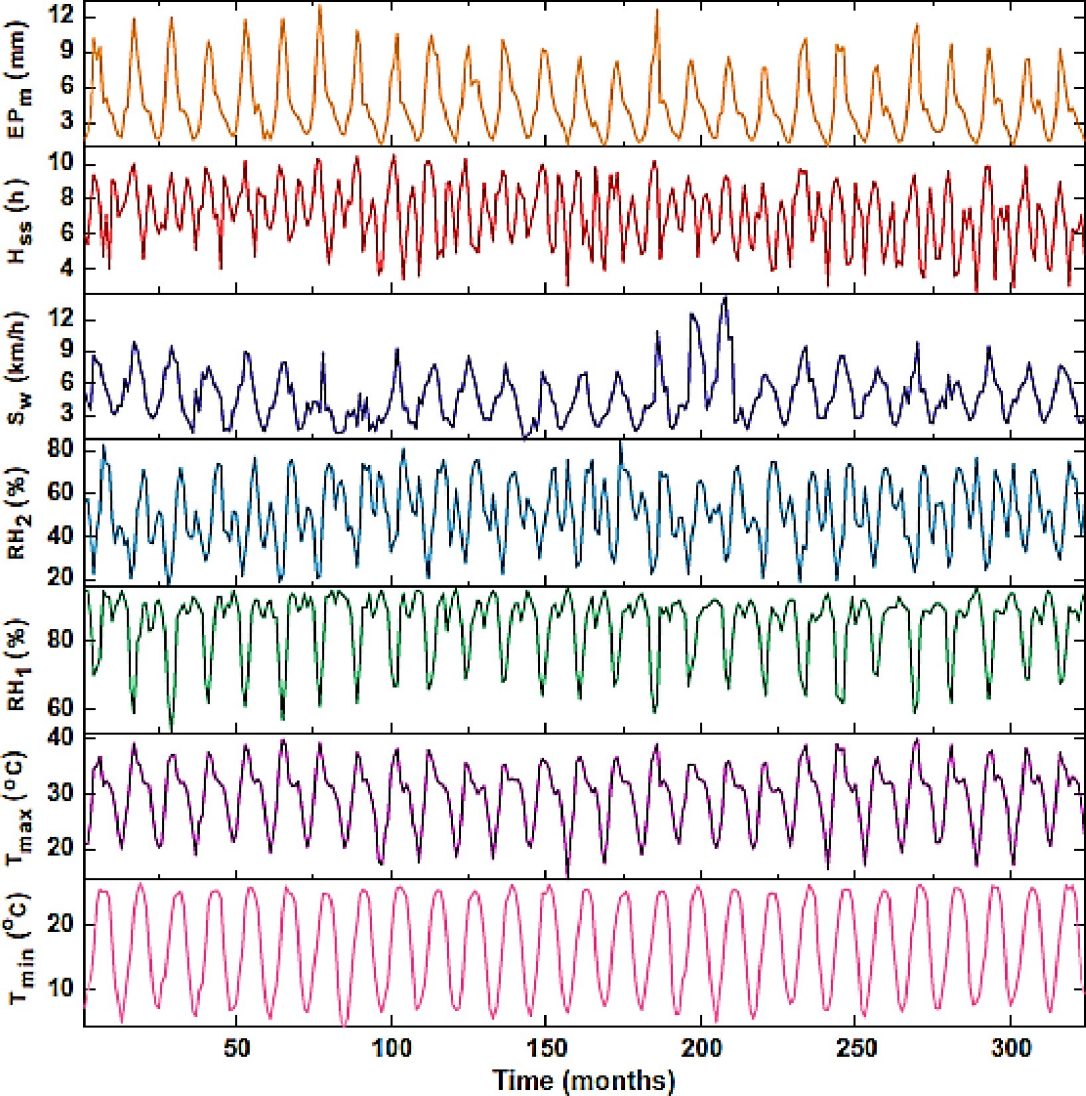
Variation of different parameters for the basin.

Fig 7 shows information about the basin data. Information for January 1990 to December 2016 is considered in this study. The period from 1990 to 2008 was used for the training period, the period from 2008 to 2012 was used for the validation period, and the period from 2012 to December 2016 (end of period) was used for the test level. The input data are considered based on mentioned interval times (Fig 7) are inserted into the SVM to start the simulation. The model is prepared based on the training level, and the validation and test level are then considered for the models to evaluate the ability of the model comprehensively.

The following equation was used to compute the correlation of input parameters with ET_0_:

$$\rho_{X,Y}=\frac{cov\left(X,Y\right)}{\sigma_{X}\sigma_{Y}}=\frac{E[\left(X-\mu_{X}\right)\left(Y-\mu_{Y}\right)}{\sigma_{X}\sigma_{Y}}$$ (21)

where cov is the covariance between the variables X with Y, $\sigma_{X}$ and $\sigma_{Y}$ are the standard deviations of variables X and Y, $\mu_{x}$ and $\mu_{Y}$ are the mean values of variables X and Y, respectively and E is the expected value. Additionally, the following equations were used to evaluate different models; the following indices have been reported as suitable indices for the hydrological simulation by different literatures [1–3,10,12,14]:

$$MAE=\frac{1}{N}\sum_{i=1}^{N}|{\left(ET_{o}\right)}_{i}-{\left(ET_{p}\right)}_{i}|$$ (22)

$$RMSE=\sqrt{\frac{1}{N}\sum_{i=1}^{N}{\left[{\left(ET_{o}\right)}_{i}-{\left(ET_{p}\right)}_{i}\right]}^{2}}$$ (23)

$$NS=1-\frac{\sum_{i=1}^{N}{\left(ET_{oi}-ET_{pi}\right)}^{2}}{\sum_{i=1}^{N}\left(ET_{oi}-E{\bar{T}}_{oi}\right)}$$ (24)

where MAE represents the mean absolute error between the simulated and observed data, RMSE represents the root-mean-square error between the simulated and observed data, NS is the Nash Sutcliffe coefficient, ${ET}_{pi}$ is the simulated ET_0_, ${ET}_{oi}$ is the FAO-56PM ET_0_ and ${E\overset{-}{T}}_{oi}$ is the average FAO-56PM ET_0_.

One of the other indices is agreement distance (d); this index varies from 0 to 1, with a high value showing a better performance of the model.

$$d=1-\frac{\sum_{i=1}^{N}{\left(ET_{oi}-ET_{pi}\right)}^{2}}{\sum_{i=1}^{N}{\left(|ET_{pi}-E{\overset{¯}{T}}_{oi}|+|ET_{oi}-E{\overset{¯}{T}}_{oi}|\right)}^{2}}$$ (25)

Table 1 shows the comprehensive information for this station. The temperatures have significant values for the station that lead to high values of ET. Additionally, the CV value for the RH_1_ has a significant value.

**Table 1:** The information for the case study.

Statistical parameters	T_min_	T_max_	RH_1_%	RH_2_%	S_w_(km/hr)	H_ss_ (h)	EP (mm/month)
Minimum	4.3	14.5	53	17	0.70	3	1
Maximum	26.5	40	96	85	14.20	10.5	12.1
Mean	16.85	29.12	84.39	5.86	4.8	7.5	4.78
σ (standard deviation)	7.12	5.61	10.22	15.69	2.5	1.7	2.81
C_v_ (coefficient of variation)	2.32	5.27	8.4	3.26	1.89	4.2	1.69

## Results and discussion

### Examination of input combinations

Table 2 shows the relationship between different inputs and ET_0_ values. The highest correlation was observed with the maximum temperature followed by the minimum temperature. The lowest correlation was observed with ${RH}_{2}$. Furthermore, the relative humidity had an inverse relationship with ET_0_, and the wind speed had a greater effect on ET_0_ than the sunshine hours.

**Table 2:** Correlation matrix of the used datasets.

Variable	T_min_ (°C)	T_max_(°C)	RH_1%_	RH_2_%	S_W_ (km/hr)	H_ss_	EP_m_ (mm/month)
T_max_	1.000						
T_max_	0.832	1.000					
RH_1_	-0.212	-0.568	1.000				
RH_2_	0.378	-0.316	0.765	1.000			
S_W_	0.396	0.625	-0.492	-0.245	1.000		
H_ss_	0.012	0.512	-0.611	-0.681	0.216	1.000	
EP_m_	0.721	0881	-.0711	-0.271	0.651	0.613	**1.000**

Thus, using Table 2 and the correlation values, the following inputs were found for different models:

$$1-\;GP_{1},M\;5T_{1},SVR-CA)\to T_{min},T_{max},RH_{1},RH_{2},S_{w},H_{ss}$$

$$2-\;GP_{2},M\;5T_{2},SVR-CA_{2})\to T_{min},T_{max},RH_{1},H_{ss}$$

$$3-\;GP_{3},M\;5T_{3},SVR-CA_{3})\to T_{min},T_{max},S_{w},RH_{1}$$

$$4-\;GP_{4},M\;5T_{4},SVR-CA_{4})\to T_{min},T_{max}$$

### Sensitivity analysis of algorithms and different models

Similar to other evolutionary algorithms, CA has random parameters with exact values required for sensitivity analysis. The optimization algorithms contain details that should be determined based on accurate values. For example, when decision variables such as the unknown values of the SVM parameters are inserted into the algorithm, the size of the initial population of members in the algorithms should be computed. Each member includes a value for the decision variables. In the current study, each egg includes a value for the unknown parameters of the SVM. Many studies considered the value of the parameters of the optimization algorithm based on literature reviews, which is a random process, but the current article considers all probable intervals for the random parameters of CA to prepare the method for the optimization problem based on Table 3. Parameters such as ω and population size are unknown for the method, and accurate values of these parameters should be computed based on computation of the variation of the objective function versus the variation of the parameter values.

Sensitivity analysis involves examination of changes in the objective function against changes in the value of a parameter. Given this goal, the objective function of this study is to minimize the RMSE, and the parameter value that minimizes the objective function is reported as the best parameter value. For example, if we consider one combination, then the most appropriate size for the community is 30 because the value of the objective function has the lowest value. The maximum number of cuckoo eggs and the minimum number of cuckoo eggs were five and three, respectively, because the objective function for the values listed was the smallest. Moreover, the ω value for the first combination was 0.5. Other parameters and values reported for the CA for other combinations are shown in Table 3.

**Table 3:** Sensitivity analysis for CA.

First input combination							
Population size	Objective function	Maximum number of eggs	Objective function	Minimum number of eggs	Objective function	ω	Objective function
10	1.111	3	1.241	1	1.111	0.300	1.231
30	0.981	5	0.981	2	0.999	0.500	0.981
50	1.212	7	1.112	3	0.981	0.700	0.999
70	1.321	9	1.114	4	1.110	0.900	1.141
Second input combination							
10	1.565	3	1.231	1	1.456	0.30	1.345
30	1.112	5	1.112	2	1.312	0.500	1.112
50	1.121	7	1.118	3	1.112	0.700	1.116
70	1.234	9	1.124	4	1.118	0.900	1.121
Third input combination							
10	1.341	3	1.281	1	1.487	0.30	1.312
30	1.009	5	1.009	2	1.231	0.500	1.009
50	1.114	7	1.112	3	1.009	0.700	1.114
70	1.118	9	1.116	4	1.118	0.900	1.118
Fourth input combination							
10	1.445	3	1.381	1	1.389	0.30	1.376
30	1.115	5	1.115	2	1.115	0.500	1.115
50	1.121	7	1.124	3	1.128	0.700	1.129
70	1.123	9	1.261	4	1.131	0.900	1.132

### Analysis of different models

Table 4 shows the performances of different models in the training, validation and test periods. Different models of SVM-CA show the superior performance of the SVM-CA1 model with $T_{min},T_{max},{RH}_{1},{RH}_{2},S_{w},H_{ss}$ as inputs. This model reduced the RMSE values of the training, validation, and testing sets by 0.1–7%, 0.2–8% and 2–10%, respectively, compared with the SVM-CA2, SVM-CA3, and SVM-CA2 models. A comparison of SVM-CA2 and SVM-CA3 models shows that although the two models are equal in terms of the number of input parameters, the SVM-CA3 model displays better performance than the SVR-CA2 model in the training, validation and test modes because S_W_ has a higher correlation with EP_m_ compared with H_ss_. The current study applied Gaussian, trapezoidal and bell membership functions (MF) for the ANFIS models. The values of RMSE, MAE and NSE show that the ANFIS with the Gaussian membership function performs the best in the training, validation and testing modes, as shown in Table 4.

**Table 4:** Computation of ET_0_ by different models for the station.

Model	Training			Validation			Test		
	RMSE	MAE	NS	RMSE	MAE	NSE	RMSE	MAE	NSE
**SVM-CA1**	0.712	0.687	0.98	0.716	0.689	0.97	0.714	0.691	0.96
ε=0.005,c=42,γ=1.12 SVM-CA2	0.744	0.722	0.96	0.749	0.729	0.95	0.751	0.732	0.94
ε=0.005,c=47,γ=1.20 SVM-CA3	0.723	0.712	0.97	0.715	0.717	0.96	0.725	0.719	0.95
ε=0.007,c=39,γ=1.24 SVM-CA4 ε=0.005,c=40,γ=1.24	0.767	0.754	0.93	0.777	0.761	0.92	0.789	0.777	0.91
ANFIS (1) (MF: Gaussian, ONF:7)	0.733	0.689	0.97	0.737	0.697	0.96	0.747	0.723	0.95
ANFIS (2) (MF: Gaussian, ONF:7)	0.762	0.723	0.96	0.763	0.731	0.95	0.783	0.718	0.92
ANFIS (3) (MF: Gaussian, ONF:7)	0.740	0.710	0.95	0.747	0.16	0.94	0.765	0.749	0.90
ANFIS (4) (MF: Gaussian, ONF:7) ONF: Optimal number of functions	0.810	0.711	0.94	0.812	0.811	0.92	0.842	0.830	0.91
**GP1**	0.734	0.691	0.96	0.739	0.698	0.95	0.749	0.721	0.94
GP2	0.764	0.725	0.93	0.776	0.735	0.92	0.789	0.767	0.91
GP3	0.745	0.714	0.94	0.749	0.720	0.93	0.777	0.754	0.92
GP4	0.812	0.800	0.92	0.815	0.814	0.91	0.844	0.832	0.90
**M5T1**	0.789	0.721	0.94	0.791	0.746	0.93	0.811	0.789	0.92
M5T2	0.823	0.749	0.92	0.832	0.778	0.90	0.834	0.811	0.89
M5T3	0.815	0.734	0.93	0.819	0.745	0.92	0.819	0.799	0.91
M5T4	0.921	0.894	0.91	0.934	0.899	0.89	0.921	0.855	0.88
Empirical models									
${ET}_{0-PT}$	0.937	0.911	0.88	0.941	0.954	0.90	0.959	0.957	0.5
${ET}_{0-MH}$	0.935	0.899	0.89	0.939	0.935	0.89	0.951	0.940	0.86
${ET}_{0-H}$	0.923	0.896	0.90	0.937	0.923	0.88	0.949	0.941	0.87
Extracted equations for GP									
$ET_{0}=2.12\frac{T_{max}}{1.25T_{max}}+\frac{\ln(RH_{1)}}{0.25RH_{2}}+\cos\left(2.25S_{w}\right)+1.21H_{ss}\leftarrow GP_{1}$									
$ET_{0}=2.11\sqrt[{3}]{\sin T_{max}}+1.11arctan\left(\frac{1.12}{T_{min}}\right)+\cos\left(1.11RH_{1}+H_{ss}\leftarrow GP_{2}\right)$									
$ET_{0}=2.11T_{max}+1.11\ln T_{min}+RH+\cos\left(1.25S_{w}\right)\leftarrow GP_{3}$									
$ET_{0}=1.12T_{max}+\text{arctan}\left(2.25T_{min}\right)\leftarrow GP_{4}$									

For training, validation and testing, ANFIS (1) decreases RMSE and MAE by 1–14% and 3–17%, respectively, compared with other ANFIS models. This result indicates the superior performance of the ANFIS (1) model. Compared with the SVM-CA1 model, the RMSE and MAE values of ANFIS decreased by 7–15% and 5–18%, respectively, using SVM-CA, which indicates the superiority of the SVM-CA model. The ANFIS model shows superior performance over the GP and M5T models due to its lower RMSE and MAE values. Although ANFIS (2) and ANFIS (3) have the same number of parameters, ANFIS (3) performs better than ANFIS (2) with parameter S_w_. The literature shows that maximum temperature, minimum temperature and wind speed are the key parameters for the computation of ET_0_ [21–26].

Among the GP models, GP (1) with all possible inputs presents the best performance. The MAE for GP (1) dropped below 4–9%, 3–15% and 5–14% in all three stages of training, validation and testing, respectively, compared with other GP models. A comparison of the GP and SVM-CA models shows that the SVM-CA has better performance. For example, SVM-CA reduced the RMSE and MAE by 5–15% and 5–17%, respectively, in the test stage compared with GP. The weakest performance among the GP models occurred for GP (4) with inputs of only two parameters. The results for the GP model show that the increase in inputs positively affects the estimation of potential ET_0_. However, GP (2) and GP (3) have the same number of parameters, but GP (3) has a more effective S_w_ parameter than GP (2). The GP models perform better than the M5T models. For example, the RMSE of the GP models decreases from 20% to 8% for training compared with the M5T models. The same is also true for the validation and testing stages.

The results of the M5T models show that the M5T1 performs better than the other M5T models and has greater NSE coefficients for training, validation and testing than the other M5T models. For example, the RMSE is reduced by 3–15%, 2–20% and 1–12% using M5T1 compared with the other M5T models for the training, validation, and testing stages, respectively. Comparison of M5T and SVM-CA shows that the SVM-CA has a more favourable performance, e.g., the RMSE and MAE for the SVM-CA model in the test stage are 12–21% and 10–22% lower than those for the M5T model. A comparison of the GP and M5T models shows the superiority of the GP model over the M5T. Table 4 shows that the ${ET}_{0-H}$ model performs better than the other experimental models, with the RMSE and MAE in testing reduced by 0.2% and 1.5%, respectively. A comparison of the SVM-CA with the empirical models shows that the RMSE and MAE are decreased by 23–25% and 24–27%, respectively.

**Fig 8:**
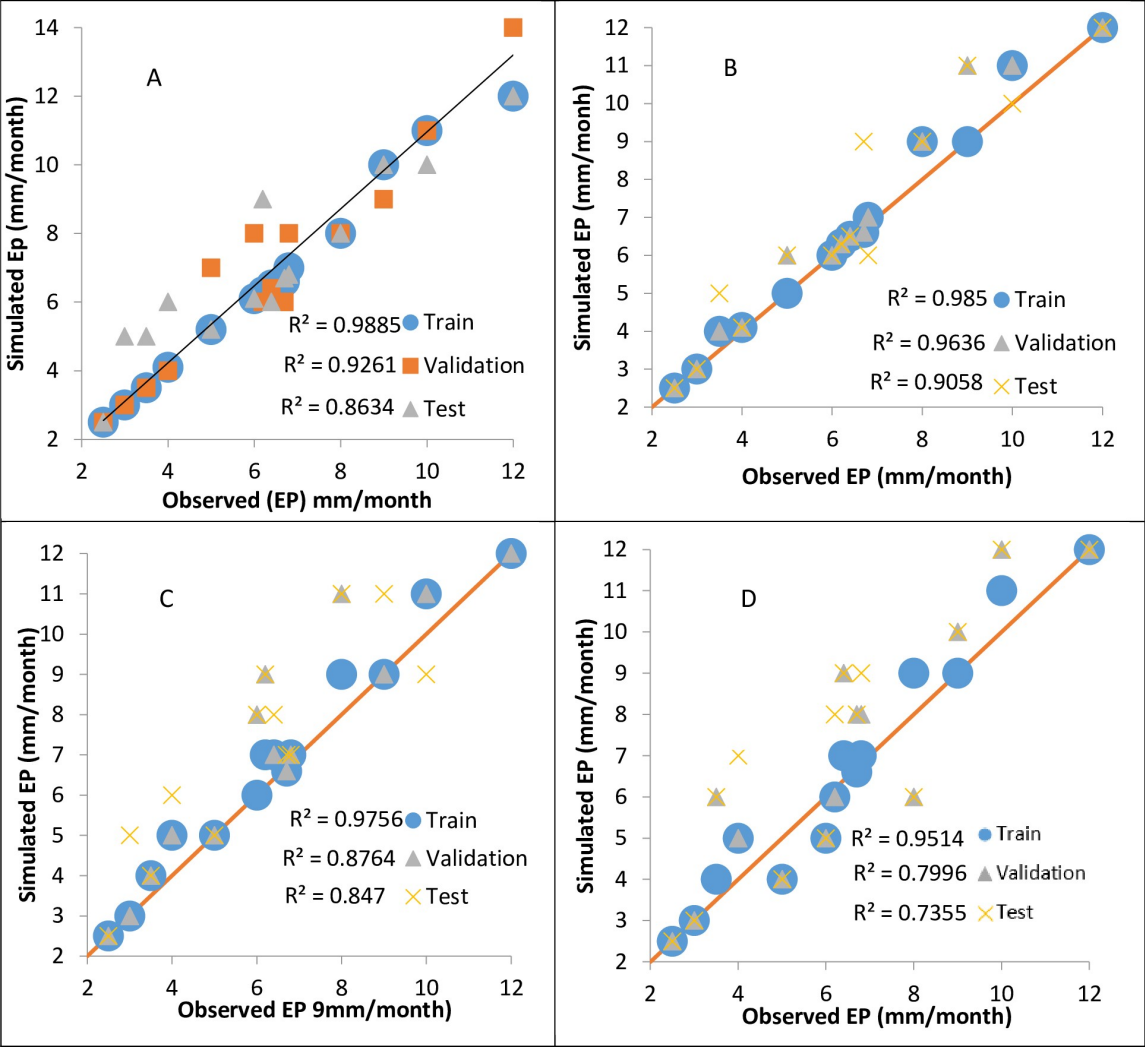
R^2^ coefficient for: (a) SVM-CA1; (b) ANFIS (1); (c) GP (1); and (d) M5T (1).

Fig 8 shows the scatterplots for the superior models, SVM-CA (1), GP (1), and M5T (1) ANFIS (1), which indicate that the SVM-CA1 has a larger R^2^ value than the other models. The SVM-CA model has less scattered estimates than the other models in the test mode. The R^2^ values for all methods are larger in the training mode than in the validation and testing modes. Malik et al. [8] used the radial neural network method and the self-organized future map neural network for the same station used in the current study. Their study indicated that the first input was the best combination for the simulation of ET_0_. The reported RMSEs for the radial neural network and self-organized future map neural network in the test stage were 1.126 and 0.718, respectively [8]. In the current study, the RMSE value for the SVM-CA1 model is 0.714, and thus the performance of the proposed model is better than the neural networks applied by Malik et al. [8]. A comparison of R^2^ for SVM-CA1 with that of a multi-layer neural network and self-organized future map neural network shows that it is equal to 0.9885 for SVM-CA1, whereas Malik et al. [8] reported values of 0.949 and 0.945.

**Fig 9:**
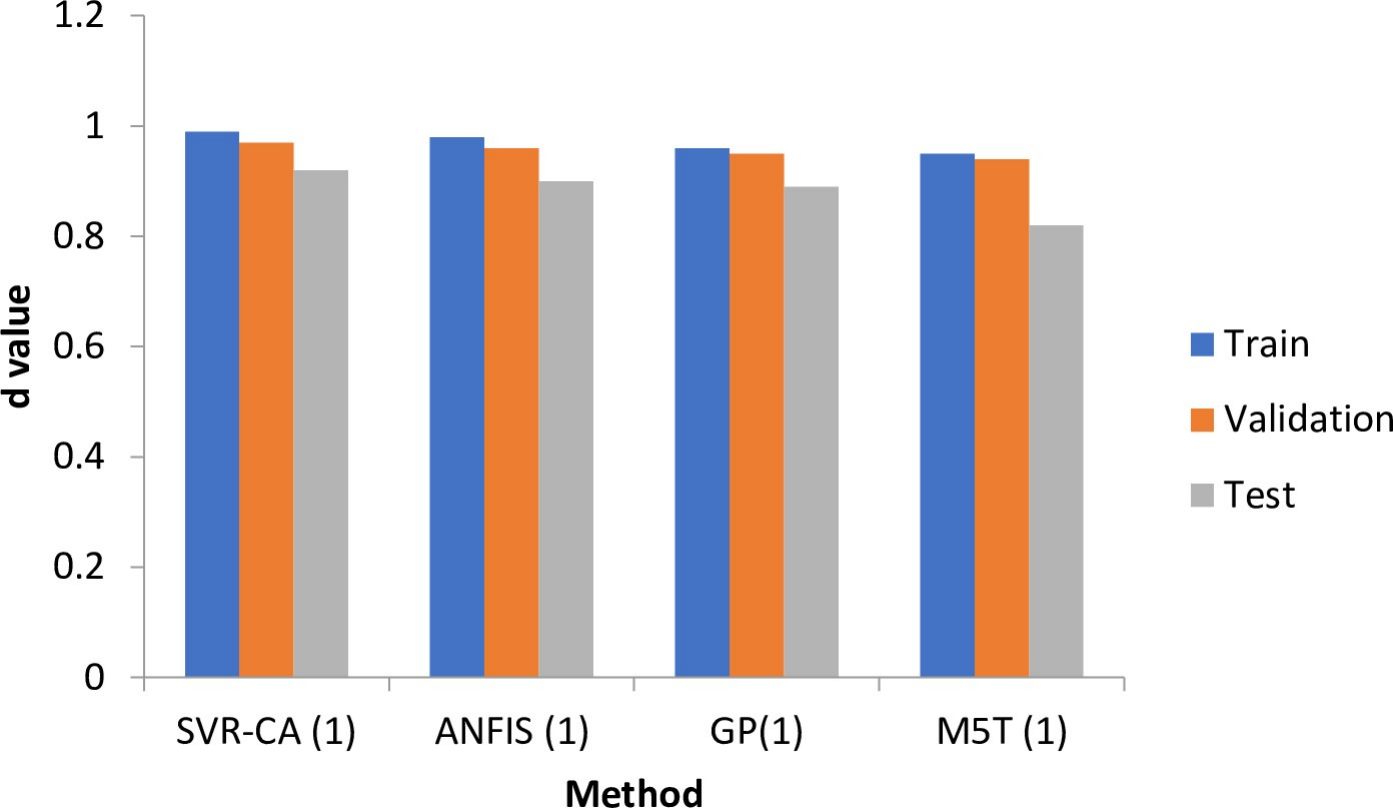
Agreement distance (d) index for different methods.

Fig 9 shows the performances of different models based on the agreement distance (d) index, which indicates that SVM-CA has the highest values for the training, validation and testing, followed by the ANFIS in ET_0_ simulation.

Table 5 compares the results with the literature reviews for this case study. The mentioned literature reviews simulated the ET_o_ based on different methods. The period of study in the mentioned literature review was January 1990 to December 2016. The co-active neuro-fuzzy inference system (CANFIS), multi-layer perceptron neural network (MLPNN) and radial basis neural network (RBNN) were considered for the simulation. The results indicated that the SVM-CA1 could decrease RMSE by 35%, 36% and 41% compared with CANFIS, MLPNN and RBNN, respectively. Additionally, the comparison of results based on NSE shows that the SVM-CA produces better results than CANFIS, MLPNN and RBNN. Furthermore, the R^2^ value for the SVM-CA1 shows better performance compared with the other methods.

**Table 5:** Comparison of results with the literature reviews for the testing level.

Index	50% total available data was used in the testing model			SVM-CA1 Present article
	CANFIS [8]	MLPNN [8]	RBNN [8]	
RMSE (mm)	1.112	1.123	1.126	0.714
NSE	0.812	0.812	0.807	0.96
R^2^	0.9212	0.9054	0.913	0.9444

One point is related to the accuracy of the results for the different methods, and other points indicate the method of preparing different methods. ANFIS can receive many inputs and has a high capability, but accurate determination of the structure of the methods, such as the number of neurons or type of membership function, is complex. The SVM has a simple structure that can be used to improve the results of the method based on processes such as the optimization algorithm. The relationship for GP showed that the method based on its ability to include different mathematical operators extracted the four different models for different inputs, but it should be noted that the crossover and mutation rate or population size should be determined for each input group. Thus, selection of accurate values for the crossover and mutation rate is complex, which can affect the accuracy of results. All three methods based on higher accuracy and accessibility of the data show better performance than the empirical models. Models such as AFIS, GP and SVM have adjustable parameters such that accurate determination of the architecture of ANFIS is complex, and therefore, these methods are effective when no simple mathematical model is available for simulation of nonlinear computations. The application of ANFIS or a more complex method is not a good choice if an easy and solvable method is available for the ET_0_ simulation.

When the Hargreaves model gives better accuracy than the two other methods, it means that the temperature parameter is highly important and effective for the simulation because this model is based on mean temperature. However, three empirical weaknesses can be observed: (1) the models can be accurate for a limited region; 2) the measurement of input data can be difficult for certain areas, and soft computing methods can simulate the results with fewer inputs; and 3) it is difficult to compare the methods because of method-specific method variables. Additionally, when the ANFIS, SVM-CA or tree model presents the final simulation, they are comprehensive because they consider the effect of different parameters on the ET_0_ simulation, whereas the empirical models, such as the Hargreaves model or other empirical models, are allocated to one or a limited number such as temperature or sunshine, and do not consider large input data for the simulation. Finally, the authors should emphasize that the new method is not limited to a specific region, as in empirical models, because the application of the optimization algorithm for the SVM allows the users to prepare the method for different inputs for different regions. The new method can also be used in other fields in hydrological problems. Finally, an important factor is related to the computation time. A PC computer system with an i5 CPU 2.4 GHZ processor and 500 GB RAM was used in the ET_0_ simulation. The computational times for the SVM-CA were 35 s, and 89 and 78 s for the ANN and tree models, respectively. Thus, the decision maker can consider the different factors for the application of different methods.

**Fig 10:**
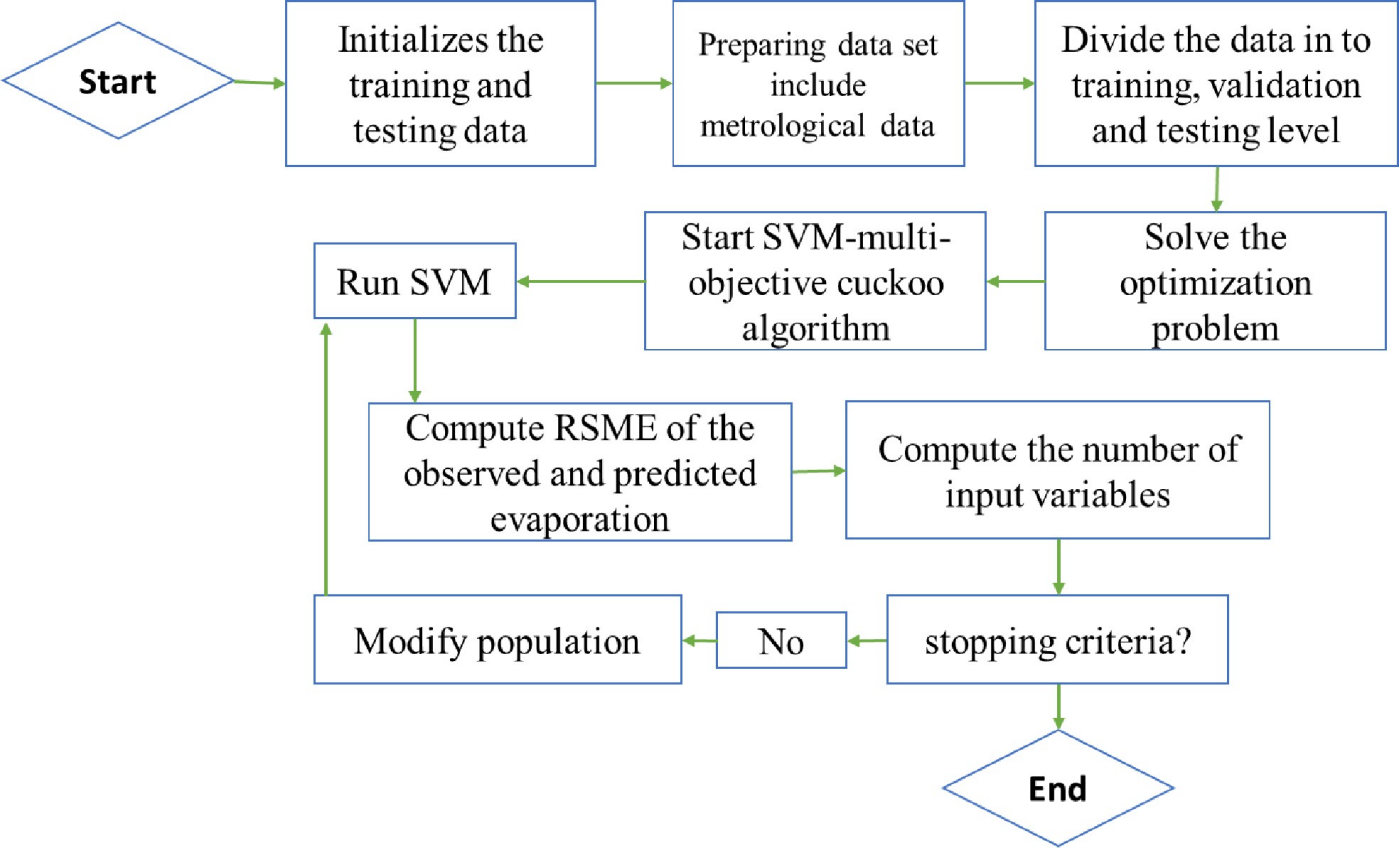
SVM-MCA structure.

The SVM-CA model can be advanced such that the method does not use Eq 20 and correlation coefficient can simulate ET. First, a multi-objective CA (MCA) can be defined based on the available literature [26,27], and SVM-MCA is considered with the dataset such that the name of the input variables and unknown SVM parameters are considered as decision variables. The aim of the problem is minimizing the number of input variables and error of the SVM for ET_0_ simulation. The decision variables are the names of the input parameters and parameters of SVM and are inserted into the algorithm as the initial population of the algorithm. When the decision variables are generated, the SVM runs, and the objective function is computed for each population member. The decision variables in each loop and iteration are modified based on defined levels of MCA, and the SVM simulates the ET_0_ based on the corrected decision variables. The process is considered until the stopping criteria are satisfied for the decision maker (Fig 10).

## Conclusion

One of the major keys to constructing adequate plans for agricultural water and irrigation management is accurate estimation of ET_0_. The current study presents a new method based on SVM and CA for the simulation of monthly ET_0_. To assess the performance of the proposed simulation model, the model was examined using ET_0_ data from India. Different scenarios for the simulation model were investigated with different alternative combinations of maximum temperature, minimum temperature, relative humidity, wind speed and sunshine hours. The results showed that the proposed SVM-CA model was more accurate than the GP, ANFIS and M5T models for simulating ET_0_. The proposed ET_0_ simulation model based on the SVM-CA method successfully reduced the RMSE and MAE by 5–15% and 5–17%, respectively, in the testing stage compared with GP. Furthermore, the study found that the best scenario combination included $T_{min},T_{max},{RH}_{1},{RH}_{2},S_{w,}H_{ss}$ as inputs, which resulted in superior performance over all other models. The worst scenario performance considered only maximum and minimum temperatures as inputs. The M5T model supplied the worst performance out of all the models, and as a result, is not recommended for simulating ET_0_.

A comparison analysis of the experimental models with SVM-CA showed that SVM-CA was more accurate, with a lower RMSE and MAE. Moreover, sensitivity analyses were conducted to evaluate the most effective input variable for the estimation of ET_0_. The model with four parameter input combinations indicated that the S_w_ parameter was a more essential variable in simulation of ET_0_ than the H_ss_ parameter. Future studies should focus on developing the SVM based on new evolutionary algorithms and using a variety of kernel functions to develop more accurate simulation models.
